# Molecular and hematological characteristics of two different δ-globin promoter variants, δ^−276(A>G)^ and δ^−77(T >C)^ among Thai, Burmese, and Laotian subjects

**DOI:** 10.7717/peerj.19636

**Published:** 2025-07-03

**Authors:** Sitthichai Panyasai, Patcharawadee Prayalaw, Kritsada Singha, Supan Fucharoen

**Affiliations:** 1 School of Allied Health Sciences, University of Phayao, Muang Phayao, Phayao, Thailand; 2 Faculty of Medical Technology, Prince of Songkla University, Muang, Songkhla, Thailand; 3 Biomedical Science Research Unit, Faculty of Medicine, Mahasarakham University, Muang, Mahasarakham, Thailand; 4 Centre for Research and Development of Medical Diagnostic Laboratories, Faculty of Associated Medical Sciences, Khon Kaen University, Muang Khon Kaen, Khon Kaen, Thailand

**Keywords:** Delta-globin gene, δ^−77(T >C)^, δ^−276(A>G)^, Genetic origin, Molecular diagnosis

## Abstract

**Background:**

We described molecular characteristics, phenotypic expression, and genetic origins of known δ^−77(T >C)^ and hitherto undescribed δ^−276(A>G)^ variants in both heterozygotes and homozygotes found in Thai, Burmese, and Laotian subjects.

**Methods:**

A family and 19 unrelated subjects with absent or decreased hemoglobin (Hb) A_2_ levels referred to three thalassemia diagnostic centers in the north, northeast, and south of Thailand were recruited. Hematological parameters were recorded, and Hb analysis was done using capillary electrophoresis. Molecular analysis of globin genes was carried out by PCR-based methods. β-Globin gene haplotype analysis, including seven DNA polymorphic sites, was done using the PCR-RFLP assay, and the results were compared with those described in the Japanese subject.

**Results:**

A proband with Hb E trait and decreased Hb A_2_ was identified. DNA sequencing of the δ-globin gene revealed a heterozygosity for a hitherto undescribed δ^−276(A>G)^ transition. However, unusually decreased Hb A_2_ was not observed in her family members with this δ^−276(A>G)^ mutation in both heterozygote and homozygote states. Further screening of this variant in unrelated Thai individuals revealed a high allele frequency of δ^−276(A>G)^ in the Thai population, the data indicating a non-pathological DNA polymorphism. In contrast, many Thai, Burmese, and Laotian subjects were encountered with another δ-globin promoter variant, the δ^−77(T >C)^ mutation in both heterozygote and homozygote. Most of them had normal hematological features, but decreased Hb A_2_ in heterozygotes and absent Hb A_2_ in homozygotes. β-Globin gene haplotype analysis points to different origins of this pathologic variant among Thai, Laotian, Burmese, and Japanese populations.

**Conclusions:**

This study described the molecular characteristics and phenotype-genotype correlation of two different δ-globin promoter variants, δ^−77(T >C)^ and δ^−276(A>G)^, found in the Southeast Asian population. Since the level of Hb A_2_ is useful for the diagnosis of several forms of thalassemia and hemoglobinopathies, the study of the δ-globin gene in areas endemic for thalassemia and hemoglobinopathies should facilitate a prevention and control program of the diseases in the region.

## Introduction

Normal adult human hemoglobin (Hb) after one year of age contains approximately 97.5% Hb A (α_2_β_2_), 2.0–3.0% Hb A_2_ (α_2_δ_2_), and 0.5% Hb F (α_2_γ_2_) (Steinberg et al., 2009). Thalassemia and hemoglobinopathies have been studied mostly on α- and β-globin genes rather than γ- and δ-globin genes because the major adult Hbs are assembled from α- and β-globin chains, and α- and β-thalassemia are more clinically significant. In contrast, δ-hemoglobinopathies, described occasionally, usually have no clinical symptoms or minor hematological changes due to Hb A_2_ expression of less than 3.5% (Steinberg et al., 2009). However, increased Hb A_2_ level is an important diagnostic marker for a β-thalassemia carrier (Soontornpanawet et al., 2023). Interactions between the δ-globin gene and β-thalassemia defects may lead to normal Hb A_2_ β-thalassemia and a misdiagnosis of β-thalassemia carrier and a pitfall in prenatal diagnosis (Mosca et al., 2009). δ-Hemoglobinopathies have been sporadically described worldwide, including, at present, 134 δ-globin chain variants and 66 δ-thalassemia mutations (accessed on January 21, 2025) (Kountouris et al., 2014). Of these, fifteen variants have been reported in Thailand (Hanart et al., 2023; Phasit et al., 2022; Prajantasen et al., 2021; Singha, Fucharoen & Fucharoen, 2021; Nuinoon et al., 2017; Panyasai, Fucharoen & Fucharoen, 2015). Only one δ-globin chain variant has been documented among Laotians (Jomoui et al., 2019), but none has been reported in Burmese. Immigration among neighboring countries creates more genetic and ethnic diversity in the regions. Here, we demonstrate molecular characteristics, phenotypic expression, and genetic origins of known and novel δ-globin promoter variants, δ^−77(T >C)^ and δ^−276(A>G)^, found in both heterozygote and homozygote states in Thai, Burmese, and Laotian individuals.

## Materials and Methods

### Subjects and hematological analysis

Ethical approval of the study protocol was obtained from the Institutional Review Board (IRB) of Khon Kaen University (HE652154), University of Phayao (1.2/026/67), and Prince of Songkla University (EC67-03). A study was done on leftover blood specimens; the IRB waived the need for informed consent from the participants. Leftover specimens of a Thai family and 19 unrelated subjects with absent or decreased Hb A_2_, referred to the three university centers for thalassemia investigation, were recruited. Sixty-seven normal Thai subjects with normal hematological parameters were also recruited as controls to study a novel δ^−276(A>G)^ variant. Hematological parameters were analyzed by standard automated blood cell counters. Hb analysis was carried out by capillary electrophoresis (Capillary 2 Flex piercing; Sebia, Lisses, France).

### Routine DNA analysis

Identifications of known α-thalassemia mutations [–^SEA^, –^THAI^, −α^3.7^, –α^4.2^, Hb Constant Spring (HBA2:c.427T>C), and Hb Paksé (HBA2:c.429A>T)] and δ-thalassemia mutations [δ^−44(G>A)^ (HBD:c.-94G>A), δ^−68(C>T)^ (HBD:c.-118C>T), δ^−77(T>C)^ (HBD:c.-127T>C), δ^A2−Troodos^ (HBD:c.349C>T), δ^IVSII- 897(A>C)^ (HBD:c.316-2A>C), and δ^CD30(AGG>GGG)^ (HBD:c.91A>G)] mutations were performed routinely using GAP-PCR and allele-specific PCR assays as described previously (Singha, Fucharoen & Fucharoen, 2021; Yamsri et al., 2010). Direct DNA sequencing of the whole δ-globin gene was done on an ABI PRISM™ 3730 XL analyzer (Applied Biosystems, Foster City, CA, USA) or Barcode-tagged sequencing (BTSeq™, Celemics, Korea). Seven DNA polymorphic sites, including *ɛ*-*Hinc* II, ^G^γ-*Hind* III, ^A^γ-*Hind* III, ψβ-*Hinc* II, 3′ψβ-*Hinc* II, β-*Ava* II, and 3′β-*BamH* I, of the β-globin gene haplotype, were analyzed by PCR-restriction fragment length polymorphism (PCR-RFLP) assays as described (Fucharoen et al., 2002).

### Development of PCR-RFLP for identification of δ^−276(*A*>*G*)^

Identification of the previously undescribed variant, δ^−276(A>G)^, was developed on a PCR-RFLP assay. Primers DT7 (5′-ATCTCTAGAGGCAAAGAAGA-3′) and F16 were used to amplify the δ-globin gene fragment with 976 bp in length. The δ^−276(A>G)^ creates a new *Xmn* I (GAANN^▾^NNTTC) restriction site. After *Xmn* I digestion (New England Biolabs, Beverly, MA, USA), the δ^−276(A>G)^ specific fragment is digested into the 839 bp and 137 bp fragments, whereas its normal counterpart without *Xmn* I restriction site remains at 976 bp in length.

### Development of allele-specific PCR for detection of the homozygous δ^−77(T>C)^

Allele-specific PCR was developed for rapid identification and confirmation of the homozygous δ^−77(T>C)^ (HBD:c.−326A>G) mutation. The reverse primer G218 (5′-GTGAGCAGGTTGGTTTAAGATA-3′) was specific to the wild-type allele of δ^−77(T>C)^. Primer pairs (G58 (5′-AGGGCAAGTTAAGGGAATAG-3′) and F16 (5′-GAGCAGGTAGGTAAAAGAAC-3′)) and [G58 and G218] were used to amplify the 713 bp and 90 bp for internal control and for homozygous δ^−77(T>C)^, respectively. The PCR reaction mixture (50 µl) contains 50–100 ng genomic DNA, 30 pmoles of all primers, 200 µM dNTPs, and 1 unit *Taq* DNA polymerase (New England Biolabs, Beverly, MA, USA) in 10 mM Tris–HCl buffer pH 8.3, 50 mM KCl, 3 mM MgCl_2_ and 0.01% gelatin. PCR amplification with an initial heat activation step at 94 °C for 3 min, followed by 30 PCR cycles of 94 °C for 1 min and 62 °C for 1 min 30 s, was carried out on a T-Personal Thermocycler (Biometra; GmbH, Gottingen, Germany). The PCR product was separated on a 1.5% agarose gel electrophoresis, stained with ethidium bromide, and visualized under UV light.

### Prediction of transcription factor binding site for δ^−276(A>G)^ and δ^−77(T>C)^

Transcription factor binding sites across the δ^−77(T>C)^ and δ^−276(A>G)^ were predicted using the online TFBIND (https://tfbind.hgc.jp/) to compare the similarity score-related transcription factors binding affinity between the wild-type and variant sequences (Tsunoda & Takagi, 1999). Similarity score (0–1) is a weight matrix in the transcription factor database TRANSFAC R.3.4 between a registered sequence for the transcription factor binding sites and the input sequence.

### *In silico* pathogenicity prediction for δ^−276(A>G)^ and δ^−77(T>C)^

Combined Annotations Dependent Depletion (CADD) 1.7 score on UCSC Genome Browser on Human (GRCh38/hg38) was used to predict *in silico* pathogenicity (https://genome.ucsc.edu/) (Kircher et al., 2014). Higher scores are more likely to be deleterious. For example, a scaled score of ≥ 10 indicates a raw score in the top 10% of all possible reference genome SNVs, and a score of ≥ 20 indicates a raw score in the top 1%.

## Results

**Figure 1:**
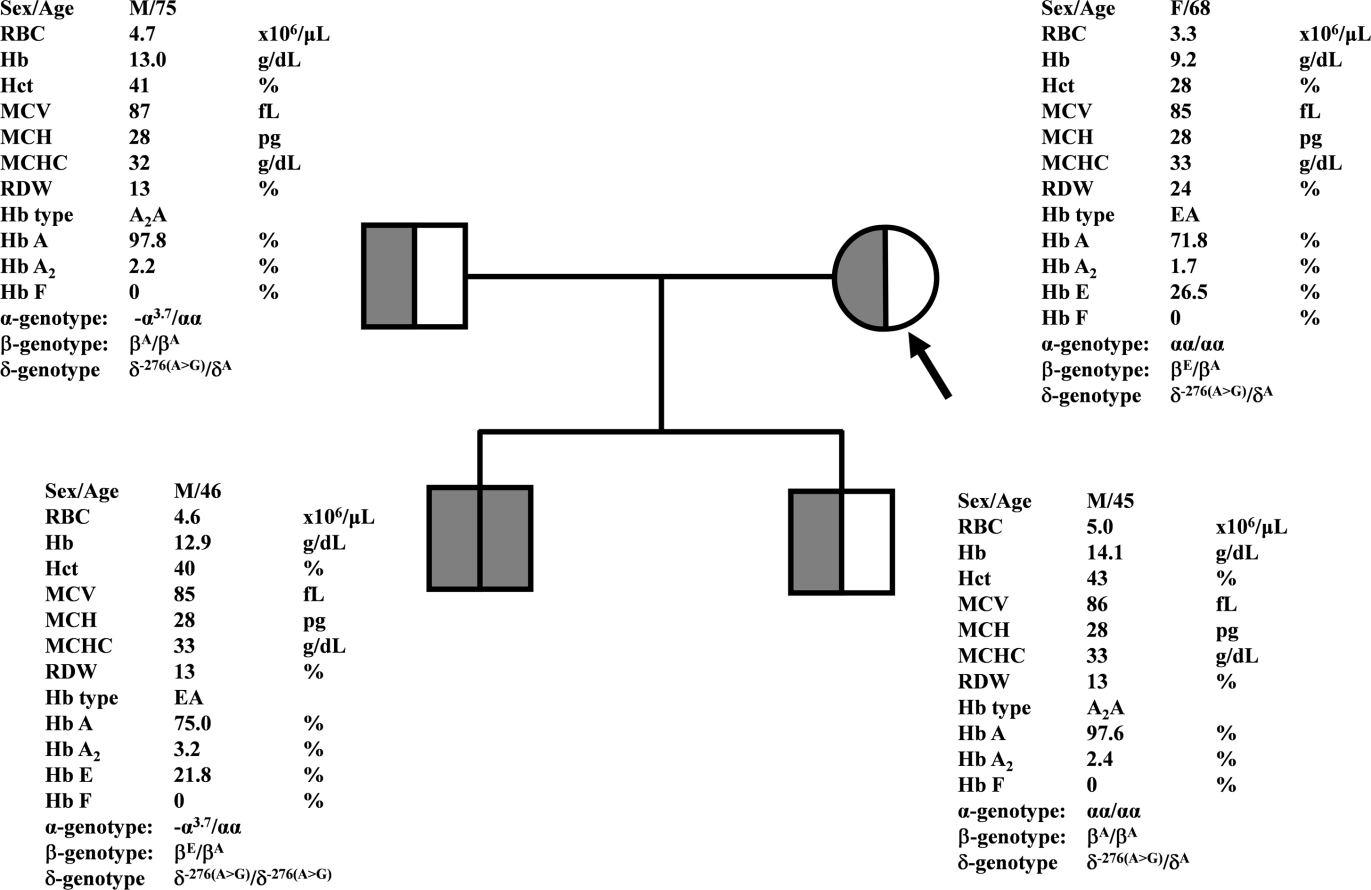
Pedigree analysis of a Thai family with δ^−276(A>G)^. The arrow indicated the proband who was heterozygous δ^−276(A>G)^ with decreased Hb A_2_ level. Her husband and the second son were also heterozygous δ^−276(A>G)^ but with normal Hb A_2_ levels. Her first son was homozygous δ^−276(A>G)^ with normal Hb A_2_ level.

**Figure 2:**
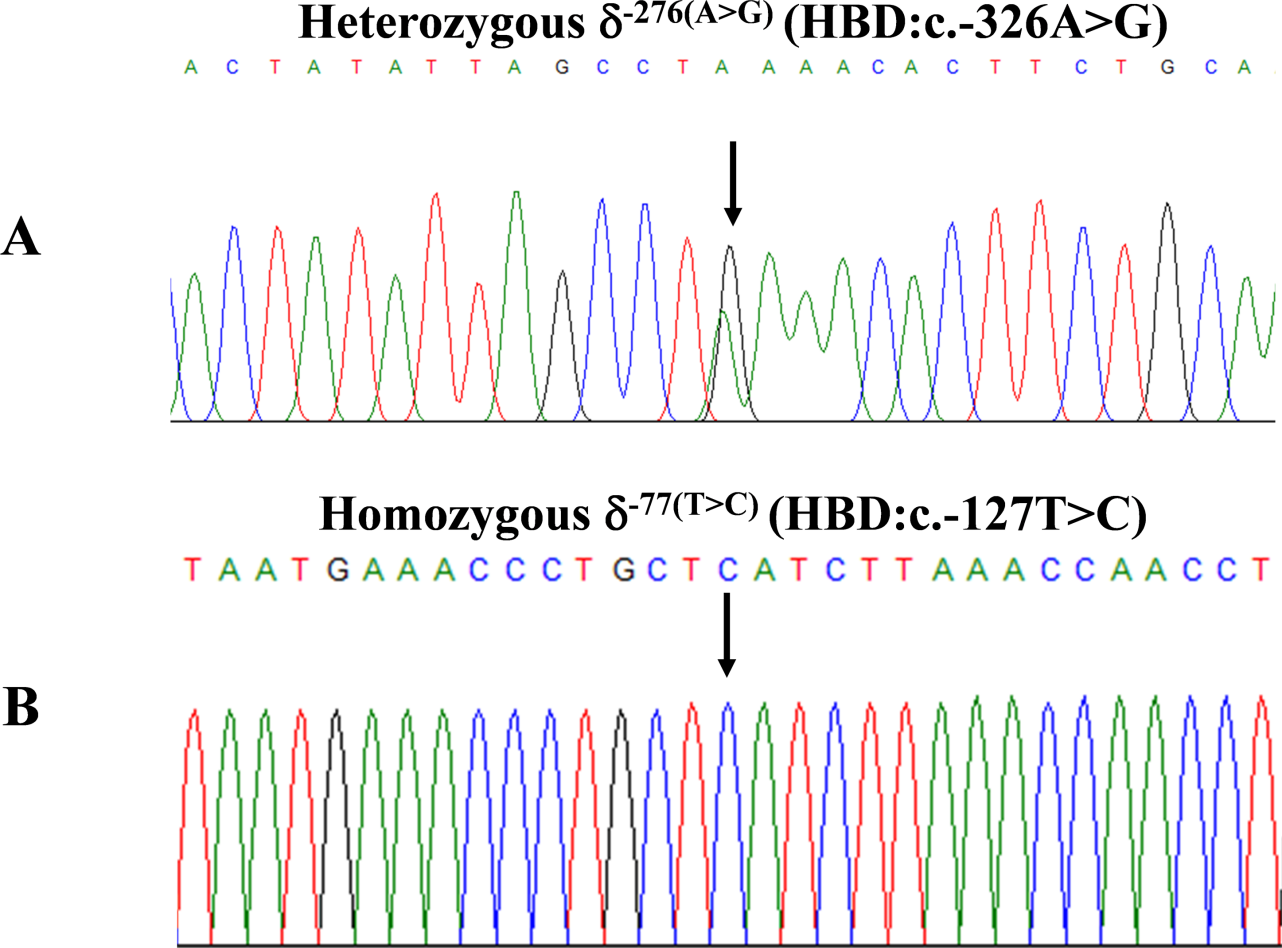
DNA sequencing profiles of the heterozygous δ^−276(A>G)^ (A) and the homozygous δ^−77(T>C)^ (B).

**Figure 3:**
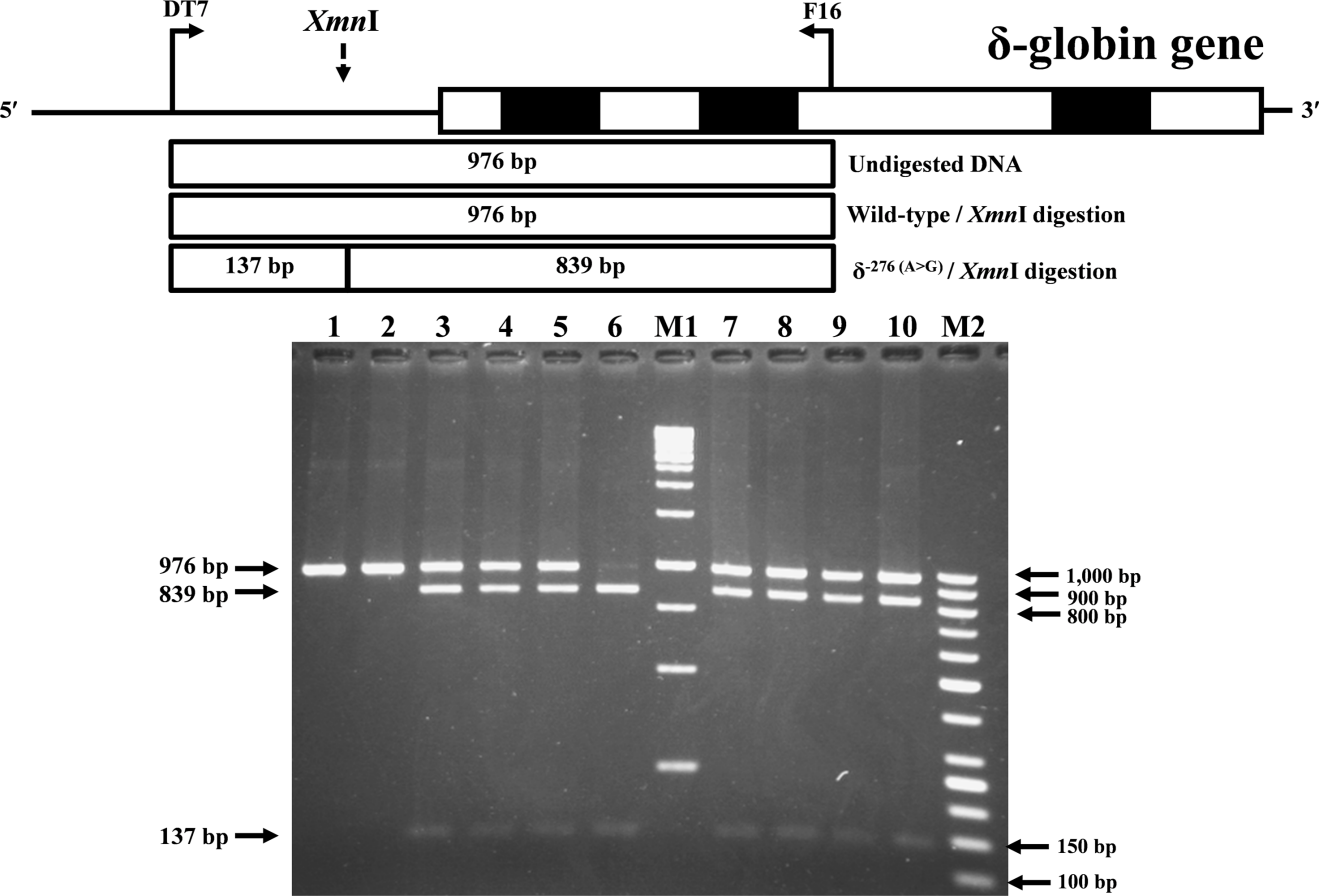
Identification of δ^−276(A>G)^ using PCR-RFLP assay with *Xmn* I digestion. Locations and orientations of primers DT7 and F16 are indicated to produce a PCR fragment of 976 bp. The *Xmn* I-digested fragments of 976 bp for wild-type, and 839 bp & 137 bp for δ^−276(A>G)^ are depicted. M1 and M2 represent the GeneRuler TM 1 kb DNA ladder and 50 bp DNA ladder, respectively. Lane 1: undigested amplified DNA, lane 2: *Xmn* I-digested amplified DNA of wild-type, lanes 3–5: *Xmn* I-digested amplified DNA of the proband, her husband, and the second son, respectively, with heterozygous δ^−276(A>G)^, lane 6: *Xmn* I-digested amplified DNA of the first son with homozygous δ^−276(A>G)^, lanes 7–10: *Xmn* I-digested amplified DNA of subjects with heterozygous δ^−276(A>G)^.

During a routine investigation of thalassemia and hemoglobinopathies, we encountered a Thai family with unusually low Hb A_2_ levels for Hb E trait, as shown in Fig. 1. The proband was a 68-year-old woman who was an Hb E carrier with decreased Hb A_2_ level (1.7%) as compared to that generally observed for Hb E trait (3.8 ± 0.3%) (Sae-ung et al., 2012). Further DNA sequencing of the δ-globin gene identified a novel δ^−276(A>G)^ in heterozygotic form (Fig. 2A). This nucleotide change was confirmed by a PCR-RFLP assay with the restriction enzyme *XmnI*, newly developed, as shown in Fig. 3. Family analysis identified heterozygosity for the δ^−276(A>G)^ in the proband, her husband, and her second son, whereas a homozygosity for the δ^−276(A>G)^ was detected in the first son. As shown in Fig. 3, we alternatively observed normal Hb A_2_ levels in her family members, which might indicate a non-pathological variant of this mutation. This prompted us to look for this mutation in the general population. The PCR-RFLP assay was therefore used to screen for the δ^−276(A>G)^in 67 normal Thai subjects with a total of 134 chromosomes. Eight of 134 chromosomes of the alternative allele (G allele) were identified with an allele frequency of 0.0597 (5.97%). This nucleotide variant has been deposited and reported as rs3813726 (https://www.ncbi.nlm.nih.gov/snp/rs3813726). In Table 1, this database indicates that the allele frequency of an alternative G-allele is 0.027863 (2.79%) in the global, 0.0316 (3.16%) in East Asian, and 0.0921 (9.21%) in the South Asian populations.

**Figure 4:**
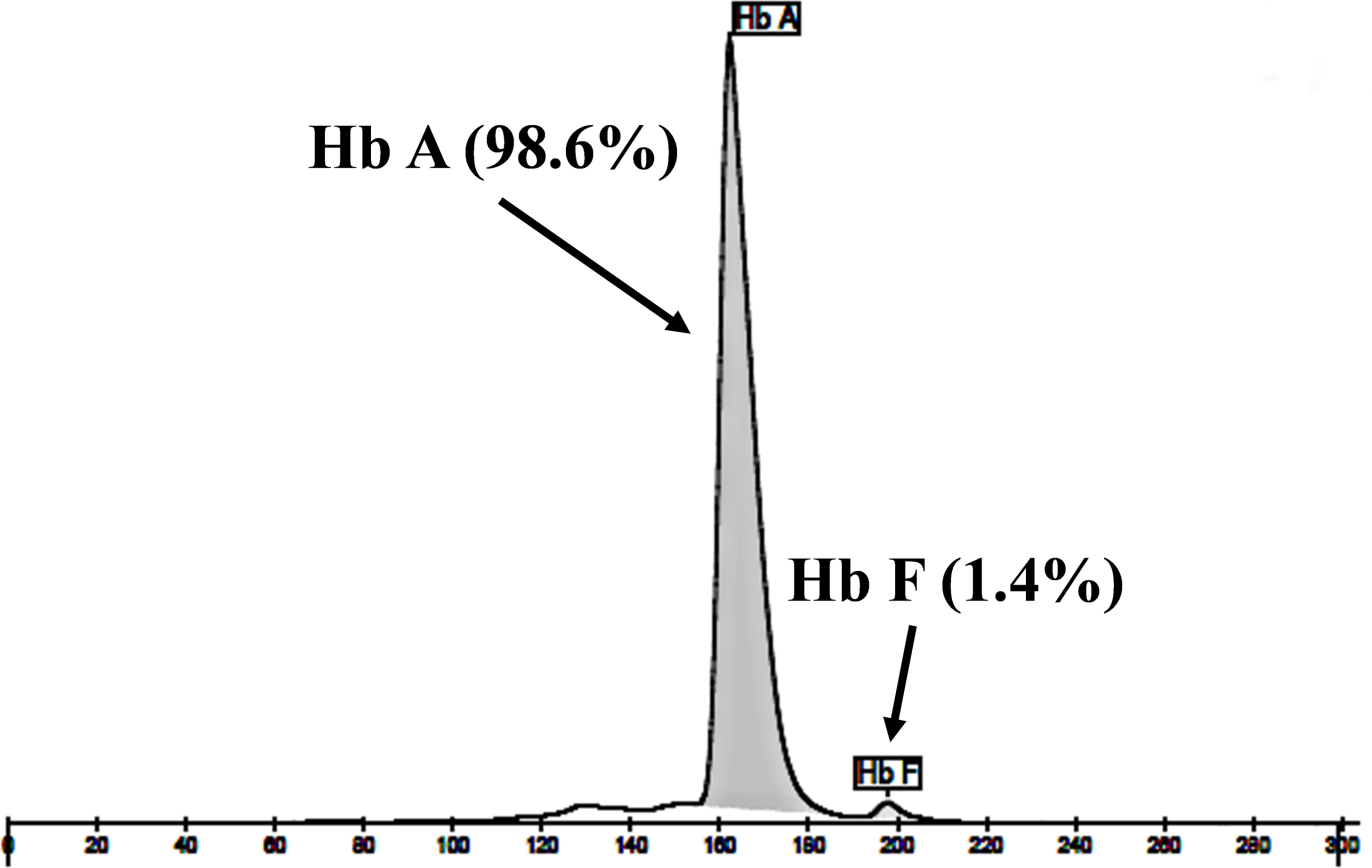
Hb analysis of the representative subject. Hb analysis using capillary electrophoresis of representative subject with homozygous δ^−77(T>C)^. Hb analysis showed only Hb A and Hb F without Hb A_2_.

**Figure 5:**
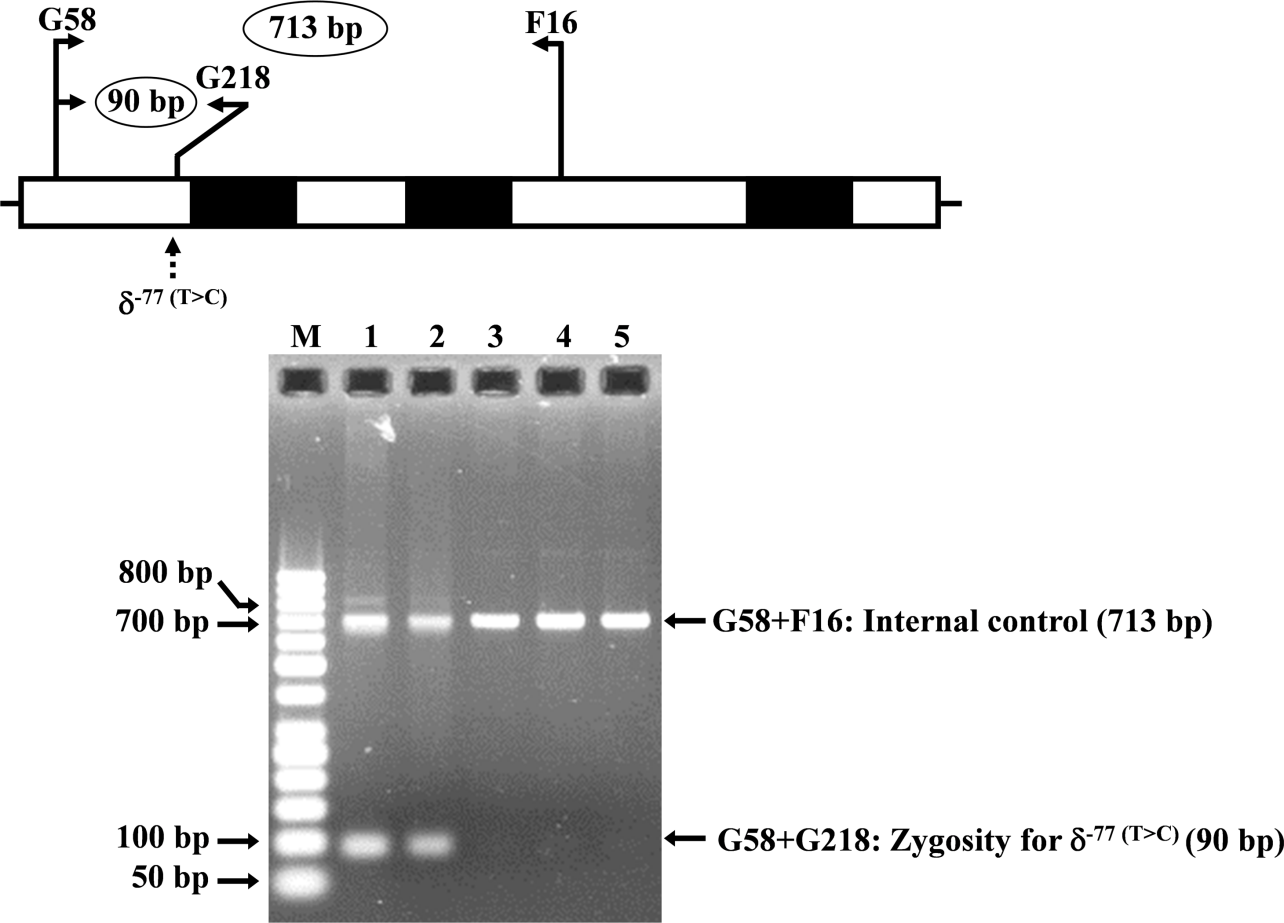
Identification of the δ^−77(T>C)^ mutation using allele-specific PCR assay as described in the Materials and Methods section. M represents the GeneRuler TM 50 bp DNA ladder. Lane 1: negative control, lane 2: heterozygous δ^−77(T>C)^, lanes 3–5: homozygous δ^−77(T>C)^.

The expanded study on 19 unrelated subjects, including 11 Thai, 6 Burmese, and 2 Laotian ethnic groups with absent or decreased Hb A_2_ levels, identified the δ^−77(T>C)^ mutation by DNA analysis. Of these 19 subjects, three subjects with totally absent Hb A_2_ (Fig. 4) were identified as homozygous δ-thalassemia (Fig. 2B). This homozygous δ^−77(T>C)^ was confirmed in allele-specific PCR assay, as shown in Fig. 5, and has been reported for the first time among the Southeast Asian population. To address the origin of this mutation among the population, β-globin gene haplotype analysis, including seven polymorphic sites within the β-globin gene cluster, was carried out. Table 2 summarizes hematological parameters, Hb analysis results, and β-globin haplotypes linked to the δ^−77(T>C)^ gene found in this study. A total of 16 subjects with 19 chromosomes could be segregated in β-globin gene haplotyping. Taking the results of β-globin gene haplotyping in the three homozygotic subjects into consideration (Table 2, subject numbers 17–19), it could be concluded that the δ^−77(T>C)^ gene identified in our subjects was linked to the β-globin gene haplotypes, [-, +, -, +, +, +, +] which is difference from that described previously in the Japanese patient, [-, +, -, +, +, -, +].

To explain the phenotype-genotype correlation of the two promoter variants, δ^−276(A>G)^ and δ^−77(T>C)^ , the online TFBIND (https://tfbind.hgc.jp/) was used to predict transcription factor binding sites by comparing the similarity score-related transcription factors binding affinity between wild-type and the variant sequences (Tables S1 and S2). In addition, the CADD 1.7 score was also used to predict *in silico* pathogenicity. The score of δ^−276(A>G)^ was 0.559, and δ^−77(T>C)^ was 1.482.

## Discussion

A decreased level or absence of Hb A_2_ and the finding of Hb A_2_ derivatives are important markers for suspecting δ-hemoglobinopathies. Decreased Hb A_2_ level at less than 1.8−2.0% has been used to screen δ-hemoglobinopathies in subjects with normal Hb type of A_2_A (Mosca et al., 2009; Singha, Fucharoen & Fucharoen, 2021; Morgado et al., 2007). It has been documented that Hb A_2_ separated from Hb E in the heterozygous Hb E on capillary electrophoresis is 3.8 ± 0.3% (Sae-ung et al., 2012). The cut-off of Hb A_2_ level <2.5% has been used to screen δ-globin gene defects in heterozygous Hb E (Singha, Fucharoen & Fucharoen, 2021). With these criteria, a δ-globin gene defect was suspected in the proband who had Hb E trait with Hb A_2_ of 1.7%. Accordingly, a novel variant, δ^−276(A>G)^, was detected in the proband and her family members (Fig. 1). However, decreased Hb A_2_ levels were not observed in the family members with δ^−276(A>G)^, in both heterozygous and homozygous δ^−276(A>G)^. This likely points to a DNA polymorphism rather than a pathological defect affecting δ-globin gene expression. In addition, the allele frequencies of the δ^−276(A>G)^ (rs3813726) were found to be 0.027863 (2.79%), 0.0316 (3.16%), 0.0921 (9.21%), and 0.0597 (5.97%) in global, East Asian, South Asian, and Thai populations, respectively (Table 1). Prediction on the effect of this δ^−276(A>G)^ mutation on the binding of transcription factors, including the TATA and CCAAT boxes (Tables S1), indicated that it might not directly affect the binding of these transcription factors to the δ-globin gene promoter. Based on this information, it is most likely that the δ^−276(A>G)^ found in this study is a relatively common non-pathological DNA polymorphism (Tamana et al., 2022). Therefore, it is likely that the decreased Hb A_2_ level (1.7%) found in the proband should not result from the δ^−276(A>G)^. It is also noteworthy that her husband and second son, who were both heterozygous for this mutation, had normal levels of Hb A_2_ (2.2−2.4%). The reduced Hb A_2_ level (1.7%) of the proband could result from another factor, such as anemia observed with her (Mosca et al., 2009). Unfortunately, the iron study and clinical data were unavailable.

**Table 1:** Allele frequencies of δ^ −276(A >G)^ (NCBI rs3813726) among healthy Thai, global, and Asian populations.

**Population**	**Number of alleles**	**NCBI rs3813726** ** (gnomAD v4 - Genome)**	
		**Reference allele (A allele)**	**Alternate allele (G allele)**
Global	149,264	0.972137 (97.21%)	0.027863 (2.79%)
East Asian	5,186	0.9684 (96.84%)	0.0316 (3.16%)
South Asian	4,822	0.9079 (90.79%)	0.0921 (9.21%)
Thai (This study)	134	0.9403 (94.03%)	0.0597 (5.97%)

In 1980, Ohta et al. (1980) described 10 homozygous δ-thalassemia with absent Hb A_2_ and 18 heterozygous δ-thalassemia with reduced Hb A_2_ levels in Japanese subjects. Some of them were further investigated by molecular analysis (Nakamura et al., 1987). The δ^−77(T>C)^ was characterized and suspected to be δ^0^-thalassemia based on the absent Hb A_2_ in the homozygotes. Further functional study showed that the δ^−77(T>C)^ disrupts the binding motif of an erythroid-specific transcription factor GATA-1 and reduces δ-globin gene expression about 20-fold as compared to normal control, leading to the δ^0^-thalassemia phenotype (Matsuda, Sakamoto & Fukumaki, 1992). Heterozygous δ^−77(T>C)^ has been later reported in Chinese, Thai, and Hong Kong Chinese (Singha, Fucharoen & Fucharoen, 2021; Liu et al., 2013; Chan et al., 2021). No homozygotic case has been documented. This mutation has now been reported for the first time in Burmese and Laotian subjects, and the homozygotic form has also been documented in Thai and Burmese subjects. As expected, most of them with δ^−77(T>C)^ in heterozygote and homozygote had normal hematological parameters. However, some subjects had anemia, which may result from other causes, such as iron deficiency anemia, commonly seen in the region. Moreover, the unusually low Hb A_2_ level (0.6%) in subject no. 7 with heterozygous δ^−77(T>C)^ could not be explained as no other mutation was observed in the whole δ-globin gene sequencing, normal α-globin gene, and mild anemia with normal other hematological parameters. Decreased Hb A_2_ levels in the heterozygote and absent Hb A_2_ in the homozygote were observed, confirming the δ^0^-thalassemia phenotype of this mutation. The prediction of the transcription factor binding site supported that the δ^−77(T>C)^ disrupts the GATA-1 binding to the δ-globin gene promoter (A/T GATA A/G) [(modification from TTATCT (AGAT AA) to TCATCT (AGATCA)] (Table S2), although a low CADD score of δ^−77(T>C)^ might be inconsistent with the δ^0^-thalassemia phenotype (Matsuda, Sakamoto & Fukumaki, 1992).

**Table 2:** Hematological parameters, Hb analysis, and *β*-globin gene haplotypes of 19 subjects with δ^−77(T>C)^ mutation in this study.

**No**	**Sex/age**	**Origin**	**RBC** **(10** ^12^ **/L)**	**Hb** **(g/dL)**	HCT **(%)**	**MCV** **(fL)**	**MCH** **(pg)**	**MCHC** **(g/dL)**	**RDW** (%)	**Hb type**	**Hb E** (%)	**Hb A**_2_ **(%)**	**Hb F** (%)	**δ-genotype**	***α*-genotype**	***β*-genotype**	*β*-**globin gene haplotype**
1	M/58	Thai	5.5	15.2	47.3	86.0	27.6	32.1	14.8	A_2_A	–	1.2	0	δ^−77(T>C)^/δ^A^	*αα*/*αα*	*β*^A^/*β*^A^	Na
2	M/45	Thai	4.6	13.4	40.3	86.9	28.8	33.2	14.2	A_2_A	–	1.8	0.8	δ^−77(T>C)^/δ^A^	*αα*/*αα*	*β*^A^/*β*^A^	[- + - + + + +]
3	F/20	Thai	4.6	11.3	35.4	77.6	24.8	31.9	17.8	A_2_A	–	1.4	0.8	δ^−77(T>C)^/δ^A^	*αα*/*αα*	*β*^A^/*β*^A^	[+/-, +/-, -, +/-, +/-, +/-, +/-]
4	M/35	Thai	5.4	14.2	43.0	79.8	26.3	32.9	14.2	A_2_A	–	1.6	0.9	δ^−77(T>C)^/δ^A^	-*α*^3.7^/*αα*	*β*^A^/*β*^A^	Na
5	M/76	Thai	4.0	12.8	37.9	94.1	31.8	33.8	12.3	A_2_A	–	1.4	1.2	δ^−77(T>C)^/δ^A^	-*α*^3.7^/*αα*	*β*^A^/*β*^A^	[- + - + + + +]
6	F/67	Thai	4.2	12.7	38.1	91.2	30.4	33.3	12.0	A_2_A	–	1.2	1.4	δ^−77(T>C)^/δ^A^	-*α*^3.7^/*αα*	*β*^A^/*β*^A^	[- + - + + + +]
7	F/adult	Thai	4.3	11.8	37.0	85.5	27.2	31.8	12.8	A_2_A	–	0.6	0	δ^−77(T>C)^/δ^A^	*αα*/*αα*	*β*^A^/*β*^A^	[+/-, +/-, -, +/-, +/-, +, +/-]
8	F/26	Burmese	4.7	13.6	44.0	93.0	28.7	30.9	12.2	A_2_A	–	1.5	3.2	δ^−77(T>C)^/δ^A^	*αα*/*αα*	*β*^A^/*β*^A^	[+/-, +/-, -, +/-, +/-, +/-, +]
9	F/25	Burmese	4.2	12.9	37.9	90.2	30.7	34	13.0	A_2_A	–	1.8	3.5	δ^−77(T>C)^/δ^A^	*αα*/*αα*	*β*^A^/*β*^A^	[+/-, +/-, -, +/-, +/-, -, +]
10	M/19	Burmese	5.3	16.1	49.8	94.1	30.4	32.3	13.6	A_2_A	–	1.6	1.5	δ^−77(T>C)^/δ^A^	*αα*/*αα*	*β*^A^/*β*^A^	[+/-, +/-, -, +/-, +/-, -, +]
11	F/35	Burmese	4.9	12.8	42.2	85.6	25.9	30.3	12.5	A_2_A	–	1.6	3.1	δ^−77(T>C)^/δ^A^	-*α*^3.7^/*αα*	*β*^A^/*β*^A^	[+/-, +/-, -, +/-, +/-, +/-, +/-]
12	F/adult	Burmese	4.1	8.2	26.1	63.8	20.1	31.5	21.0	A_2_A	–	1.2	0	δ^−77(T>C)^/δ^A^	-*α*^3.7^/*αα*	*β*^A^/*β*^A^	[+/-, +/-, -, +/-, +/-, +, +/-]
13	F/26	Laotian	4.2	11.8	37.6	89.3	28.1	31.5	12.7	A_2_A	–	1.7	1.5	δ^−77(T>C)^/δ^A^	*αα*/*αα*	*β*^A^/*β*^A^	[+/-, +/-, -, +/-, +/-, -, +]
14	F/31	Laotian	4.4	12.4	41.1	92.8	28.0	30.1	11.4	A_2_A	–	1.6	1.7	δ^−77(T>C)^/δ^A^	*αα*/*αα*	*β*^A^/*β*^A^	[+/-, +/-, -, +/-, +/-, +/-, +]
		Total	4.6 ± 0.5	12.8 ± 1.9	39.8 ± 5.7	86.4 ± 8.2	27.8 ± 3.0	32.1 ± 1.2	13.9 ± 2.6			1.4 ± 0.3	1.4 ± 1.2				
15	M/adult	Thai	5.2	15.2	43.4	83.7	29.3	35.0	13.0	EA	25.7	1.9	4.9	δ^−77(T>C)^/δ^A^	*αα*/*αα*	*β*^E^/*β*^A^	Na
16	F/31	Thai	Na	Na	Na	77.2	24.2	31.3	Na	EA	25.4	1.2	2.9	δ^−77(T>C)^/δ^A^	*αα*/*αα*	*β*^E^/*β*^A^	[+/-, +, -, +, +, +, +/-]
17	M/48	Thai	5.1	15.2	46.0	92.0	30.2	32.9	14.7	A	–	0	0	δ^−77(T>C)^/δ^−77(T>C)^	*αα*/*αα*	*β*^A^/*β*^A^	[- + - + + + +]
18	F/62	Thai	3.2	7.4	24.2	75.9	23.1	30.5	20.0	A	–	0	5.4	δ^−77(T>C)^/δ^−77(T>C)^	*αα*/*αα*	*β*^A^/*β*^A^	[- + - + + + +]
19	M/44	Burmese	5.8	12.9	41.0	71.3	22.4	31.5	16.0	A	–	0	1.4	δ^−77(T>C)^/δ^−77(T>C)^	-*α*^3.7^/-*α*^3.7^	*β*^A^/*β*^A^	[- + - + + + +]
		Total	4.7 ± 1.4	11.8 ± 4.0	37.1 ± 11.4	79.7 ± 10.9	25.2 ± 4.3	31.6 ± 1.2	16.9 ± 2.8			0	2.3 ± 2.8				

**Notes.**

Na, not available

As shown in Table 2, we found that most of the δ^−77(T>C)^ genes identified in the Southeast Asian population were linked to the β-globin gene haplotypes, [-, +, -, +, +, +, +] whereas those described in the Japanese patients were associated with two different β-globin haplotypes, including [-, +, -, +, +, -, +] (*n* = 4) and [+, +, -, +, -, -, +] (*n* = 3) (Nakamura et al., 1987). This indicates likely a multiple origin of this common δ^0^-thalassemia among the Asian population which could explain its relatively high prevalence and spread in the region.

## Conclusions

This study described a novel DNA polymorphism, δ^−276(A>G)^, and its phenotype-genotype correlation, and allele frequency in the Southeast Asian population. The study also reported for the first time, the δ^−77(T>C)^ in Laotian and Burmese and the homozygotic form in Thai and Burmese subjects. β-Globin gene haplotype analysis suggests a multiple origin of this common genetic defect among Southeast Asian and Japanese populations. Nonetheless, a study on δ-hemoglobinopathies should prove useful in the region with a high prevalence of thalassemia and hemoglobinopathies to prevent pitfalls in the misdiagnosis of the cases.

## Supplemental Information

10.7717/peerj.19636/supp-1 Supplemental Information 1 Comparison of similarity score related transcription factors binding affinity between the wild-type and δ^−276(A>G)^ sequences

## Additional Information and Declarations

### DNA Deposition

The following information was supplied regarding the deposition of DNA sequences: The sequence change is available at NCBI: rs3813726.

### Data Availability

The following information was supplied regarding data availability: Raw data are available in Fig. 1 and Table 2.
