# Supplementary material for: Molecular and hematological characteristics of two different δ-globin promoter variants, δ−276(A>G) and δ−77(T >C) among Thai, Burmese, and Laotian subjects
Source: PeerJ. 2025 Jul 3;13:e19636. doi: 10.7717/peerj.19636 (PMC12229147; doi:10.7717/peerj.19636)
Supplement: Supplemental Information 1 [file peerj-13-19636-s001.docx]

**Supplementary Table 1** Comparison of similarity score related transcription factors binding affinity between the wild-type and δ^-276 (A>G)^ sequences using the online TFBIND prediction program.

| **AC ID** | **Consensus sequence** | **Wild type** | **δ^-276 (A>G)^** |
| --- | --- | --- | --- |
| M00200 V$CAAT_C | ACCAATCANCNNGCYYSNCNCWNNT | <0.71 | 0.734317 |
| M00057 V$COMP1_01 | NNTNWKGATTGRCNRSRANMRRNN | <0.77 | 0.786946 |
| M00138 V$OCT1_04 | NNNNNNNWATGCAAATNNNWNNW | 0.781054 | <0.78 |
| M00135 V$OCT1_01 | NNNNWTATGCAAATNTNNN | 0.809424 | 0.741702 |
| M00252 V$TATA_01 | STATAAAWRNNNNNN | 0.830500 | 0.818320 |
| M00210 V$OCT_C | CTNATTTGCATAY | 0.764742 | <0.76 |
| M00116 V$CEBPA_01 | NNATTRCNNAANNN | 0.811107 | <0.80 |
| M00248 V$OCT1_07 | TNTATGNTAATT | 0.799237 | <0.79 |
| M00133 V$TST1_01 | NNKGAWTWANANTNN | 0.888932 | <0.86 |
| M00155 V$ARP1_01 | TGARCCYTTGAMCCYW | 0.750909 | <0.75 |
| M00223 V$STAT_01 | TTCCCRKAA | 0.802622 | <0.79 |
| M00025 V$ELK1_02 | NNNNCCGGAARYNN | <0.75 | 0.786654 |
| M00216 V$TATA_C | NCTATAAAAR | 0.779245 | <0.74 |
| M00032 V$CETS1P54_01 | NCMGGAWGYN | <0.81 | 0.820478 |
| M00050 V$E2F_02 | TTTSGCGC | <0.74 | 0.748403 |
| M00249 V$CHOP_01 | NNRTGCAATMCCC | <0.77 | 0.782640 |
| M00174 V$AP1_Q6 | NNTGACTCANN | <0.76 | 0.784882 |
| M00199 V$AP1_C | NTGASTCAG | <0.77 | 0.800875 |
| M00251 V$XBP1_01 | NNGNTGACGTGKNNNWT | 0.786752 | 0.779274 |
| M00160 V$SRY_02 | NWWAACAAWANN | 0.778920 | <0.76 |
| M00033 V$P300_01 | NNNRGGAGTNNNNS | 0.806067 | 0.836060 |
| M00042 V$SOX5_01 | NNAACAATNN | 0.848563 | 0.839845 |

S = C or G, W = A or T, R = A or G, Y = C or T, K = G or T, M = A or C, N = any base pair

**Supplementary Table 2** Comparison of similarity score related transcription factors binding affinity between the wild-type and δ^-77 (T>C)^ sequences using the online TFBIND prediction program. The bold sequences indicate the significant transcription factors in the δ-globin gene promoter.

| **AC ID** | **Consensus sequence** | **Wild type** | **δ^-77 (T>C)^** |
| --- | --- | --- | --- |
| M00212 V$POLY_C | CAATAAAACCYYYYKCTN | <0.74 | 0.806922 |
| M00071 V$E47_02 | NNNMRCAGGTGTTMNN | <0.71 | 0.760316 |
| M00158 V$COUP_01 | TGAMCTTTGMMCYT | <0.80 | 0.824546 |
| M00255 V$GC_01 | NRGGGGCGGGGCNK | 0.818226 | 0.827780 |
| M00272 V$P53_02 | NGRCWTGYCY | 0.810961 | 0.801876 |
| M00008 V$SP1_01 | GRGGCRGGGW | 0.801246 | 0.820215 |
| M00057 V$COMP1_01 | NNTNWKGATTGRCNRSRANMRRNN | 0.809222 | 0.849820 |
| **M00203 V$GATA_C** | **NGATAAGNMNN** | **0.874495** | **<0.83** |
| M00059 V$YY1_01 | NNNNNCCATNTWNNNWN | <0.77 | 0.776868 |
| **M00126 V$GATA1_02** | **NNNNNGATANKGNN** | **0.842500** | **<0.77** |
| **M00128 V$GATA1_04** | **NNCWGATARNNNN** | **0.937806** | **<0.81** |
| M00210 V$OCT_C | CTNATTTGCATAY | 0.765347 | <0.76 |
| M00073 V$DELTAEF1_01 | NNNCACCTNAN | <0.81 | 0.818064 |
| M00076 V$GATA2_01 | NNNGATRNNN GCTTATCTTA | 0.882273 | 0.853857 |
| M00077 V$GATA3_01 | NNGATARNG | 0.887461 | 0.833850 |
| M00278 V$LMO2COM_02 | NMGATANSG | 0.934973 | <0.79 |
| M00011 V$EVI1_06 | ACAAGATAA | 0.775619 | <0.76 |
| M00079 V$EVI1_02 | AGAYAAGATAA | 0.820568 | <0.77 |
| M00080 V$EVI1_03 | AGATAAGATAA | 0.718107 | <0.71 |
| M00223 V$STAT_01 | TTCCCRKAA | 0.802622 | <0.79 |
| M00191 V$ER_Q6 | NNARGNNANNNTGACCYNN | <0.73 | 0.731226 |
| M00253 V$CAP_01 | NCANNNNN | <0.87 | 0.905372 |

S = C or G, W = A or T, R = A or G, Y = C or T, K = G or T, M = A or C, N = any base pair
